# An emotion recognition dataset using millimeter wave radar and physiological reference signals

**DOI:** 10.1038/s41597-026-07159-6

**Published:** 2026-04-06

**Authors:** Jialong Cai, Xinyan Zhang, Yang Pan, Hui Zhou

**Affiliations:** 1 School of Automation, Nanjing University of Science and Technology, Nanjing, China; 2 Department of Rehabilitation, Nanjing Brain Hospital affiliated to Nanjing Medical University, Nanjing, China; 3 Department of Neurology, Zhongnan Hospital of Wuhan University, Wuhan, China

**Keywords:** Health care, Engineering

## Abstract

We propose an emotion recognition dataset based on millimeter-wave (mmWave) radar and physiological reference signals. Compared to conventional methods, mmWave radar could obtain vital signs in a non-contact method without privacy concerns. We used validated stimuli to induce participants’ emotions and simultaneously recorded three types of signals: mmWave signals, photoplethysmography (PPG) pulse signals, and galvanic skin response (GSR) signals. Participants used the Self-Assessment Manikin (SAM) to provide subjective emotion ratings. We collected signals and emotion rating data from 15 participants and validated the effectiveness of emotion induction and the data quality. The dataset can be used for research such as: (1) mmWave radar-based vital sign extraction; (2) comparison of emotion recognition performance across different signals; (3) multi-modal fusion for emotion recognition; (4) individual differences in emotional responses; (5) cross-subject emotion recognition, among others.

## Background & Summary

Emotion recognition, as a crucial component of affective computing, aims to determine an individual’s emotional state through behaviors or physiological signals. Emotion recognition holds vast application potential across fields such as healthcare^1^, education^2^, entertainment^3^, and transportation^4^. Accurate and efficient machine learning models are crucial for creating reliable emotion recognition systems, to develop and optimize these models, high-quality datasets are necessary, particularly for data-intensive deep learning models.

Currently, numerous emotion recognition datasets have been proposed. For example, the MAHNOB-HCI^5^ dataset includes face videos, audio signals, eye gaze data, and peripheral/central nervous system physiological signals from 27 participants. When publishing DEAP^6^ dataset, Koelstra *et al*. acquired electroencephalography (EEG), facial videos, and peripheral physiological signals from participants watching music video clips, they also proposed a novel stimulus selection method. Abadi *et al*. used the same stimuli as DEAP when constructing the DECAF^7^ dataset, with the main feature being the collection of magnetoencephalogram (MEG) data. The ASCERTAIN^8^ dataset obtains physiological signals and facial features using off-the-shelf wearable sensors. In addition, there are other emotion recognition datasets such as DREAMER^9^ and AMIGOS^10^. The developers of current emotion recognition datasets typically obtain physiological signals through wearable devices which are in contact with the body, while external expressions are captured by cameras, microphones, and other devices. However, wearing devices may cause discomfort for users and interfere with the natural expression of emotions; video and audio may raise privacy concerns in practical applications.

To address this, we use millimeter-wave (mmWave) radar to obtain physiological signals and further construct an emotion recognition dataset. The advantage of mmWave radar lies in its ability to capture minute chest wall displacements caused by breathing and heartbeat without actual contact. This process is not susceptible to environmental lighting and noise interference. Additionally, it does not collect facial or vocal data, eliminating privacy leakage risks. Emotion recognition using mmWave radar holds promise as a convenient, objective, and privacy-protective novel method for emotion recognition.

In this study, we used validated film clips as stimuli to induce emotional responses in participants. We simultaneously recorded mmWave signals, photoplethysmography (PPG) pulse signals, and galvanic skin response (GSR) signals. The PPG and GSR signals could be used to compare and validate the effectiveness of mmWave and to provide possibilities for multimodal fusion research. Participants rated the stimuli using the Self-Assessment Manikin (SAM)^11^ as ground truth labels. We collected signals and subjective emotional ratings from 15 participants. In the Technical Validation, we analyzed the data to validate the effectiveness of emotion induction and the reliability of data quality. To our knowledge, no existing publicly accessible dataset has documented the use of mmWave radar for emotion recognition. This dataset can be used for research such as: (1) mmWave radar-based vital sign extraction; (2) comparison of emotion recognition performance across different signals; (3) multi-modal fusion for emotion recognition and fusion strategy comparison; (4) individual differences in emotional responses; (5) cross-subject emotion recognition, among others.

## Methods

### Stimuli

We used 18 film clips proposed by Gabert-Quillen *et al*.^12^, which are designed to induce nine specific target emotions and have been validated as effective in eliciting emotional responses^9^. These film clips have an average duration of 199 seconds, with individual clip lengths ranging from 65 to 393 seconds. We obtained the full films with Chinese subtitles. Considering that participants are required to wear sensors, whereas participants in Gabert-Quillen *et al*.’s study^12^ did not need to, the total duration of the clips might cause fatigue among participants in this study, affecting data quality. Therefore, while ensuring that the plot of the clips remained understandable, we edited out some segments to shorten the total duration of the experiment. Compared to the original clips, the edits made to the clips in this study are shown in Table 1. The average duration of the edited film clips is 183 seconds, with individual clip durations ranging from 65 to 350 seconds. For the neutral baseline clip, we continued to use the opening sequence of *Planet Earth*^12^, though it was extended at 78 seconds, covering from 00:30 to 01:48 of the documentary. It begins with a view of Earth from space, transitions to natural landscapes, and concludes with a group of birds in flight.

**Table 1:** The edits made to the film clips of Gabert-Quillen *et al*.’s study^12^.

Film clip	Target emotion	Edit
*Modern Times*	Amusement	Final 59 s removed
*The Hangover*	Amusement	First 77 s removed
*300*	Excitement	No edit
*The Bourne Identity*	Excitement	First 19 s removed
*Remember the Titans*	Happiness	First 38 s removed
*Wall-E*	Happiness	First 17 s and final 46 s removed
*Pride and Prejudice*	Calmness	No edit
*Searching for Bobby Fischer*	Calmness	First 19 s removed
*Crash*	Anger	First 43 s removed
*Gentleman’s Agreement*	Anger	First 20 s removed
*National Lampoon’s Van Wilder*	Disgust	No edit
*The Fly*	Disgust	No edit
*Psycho*	Fear	First 82 s removed
*The Ring*	Fear	First 11 s removed
*My Girl*	Sadness	No edit
*The Shawshank Redemption*	Sadness	No edit
*D.O.A*.	Surprise	No edit
*The Departed*	Surprise	First 5 s removed

### Ethics statement

The research was approved by the Ethics Review Board of Nanjing Brain Hospital Affiliated to Nanjing Medical University, with approval number 2025-KY108-02. The materials submitted to the ethics review board included an overview of the research content, an ethics review application form, a research project implementation application form, and a participant informed consent form. In the informed consent form, participants were provided with information about the research overview, experimental procedures, and precautions, and were informed that they could withdraw from the experiment at any time without incurring any liability.

### Participants

We recruited 15 healthy college students as participants, aged between 19 and 25 years (mean = 22.60, standard deviation (SD) = 2.13). None of the participants had a history of cardiovascular disease or mental/psychological disorders. One participant was left-handed, while the rest were right-handed. All participants signed informed consent forms prior to the data collection and received a standardized cash reward disbursed via a digital payment platform after collection.

### Measures

Participants were required to rate their emotional experiences using the SAM^11^ after watching the stimuli. SAM represents emotional states graphically through Manikins, covering three scales: valence (from unpleasant/sad to pleasant/joyful), arousal (from calm/sleepy to alert/tense), and dominance (from powerless/passive to empowered/dominant). Each scale is rated from 1 (very low) to 9 (very high).

### Signal acquisition

In this study, we acquired vital signs obtained through mmWave radar, PPG and GSR sensors. Each modality is described in detail below.

#### mmWave

**Fig. 1:**
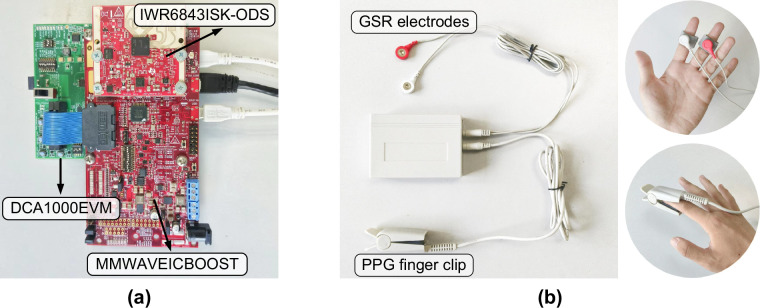
The devices used for data collection in this study. (**a**) mmWave radar. (**b**) PPG and GSR recording device.

We employed Texas Instruments (TI) IWR6843ISK-ODS mmWave sensor (https://www.ti.com/tool/IWR6843ISK-ODS), a 60–64 GHz frequency-modulated continuous wave (FMCW) radar evaluation module that transmits a linear chirp signal. Pairing with the mmWave sensor were MMWAVEICBOOST carrier card (https://www.ti.com/tool/MMWAVEICBOOST) and DCA1000EVM capture card (https://www.ti.com/tool/DCA1000EVM), where MMWAVEICBOOST provided power and connectivity for the radar module via a 60-pin HD connector; DCA1000EVM connected to the MMWAVEICBOOST to acquire raw radar data and stream it to a host computer by Ethernet using User Datagram Protocol (UDP) for subsequent processing. The mmWave radar device is shown in Fig. 1(a).

In data collection, we utilized all three transmitter (Tx) antennas and four receiver (Rx) antennas of IWR6843ISK-ODS, forming a virtual antenna array with 12 antennas through time-division multiplexing (TDM). To enhance signal quality, we applied multi-channel averaging across the 12 virtual channels, effectively leveraging the randomly distributed noise to cancel itself out, thereby enhancing the signal-to-noise ratio (SNR). The frame rate was set to 100 Hz, while the analog-to-digital converter (ADC) sampling rate is 5.12 MHz, and each chirp consists of 256 ADC samples. All radar parameters were configured and controlled using the *mmWave Studio* software (https://www.ti.com/tool/MMWAVE-STUDIO). The detailed radar parameters are shown in Table 2.

**Table 2:** Radar parameters.

Parameter	Value
Start frequency	60 GHz
Band width	3790.97 MHz
Frequency slope	66.590 MHz/μs
ADC sample rate	5.12 MHz
ADC samples number	256
Tx number	3
Rx number	4
Rx gain	30 dB
Frame rate	100 Hz
Chirp loops number	1
Idle time	7 μs
ADC start time	5.92 μs
Ramp end time	56.93 μs
Range resolution	0.0451 m

#### PPG

The PPG is an optical method for measuring volume changes of blood, which works by shining light onto the skin and detecting variations in the reflected or transmitted light caused by pulsatile blood flow. Studies have demonstrated the association between PPG signals and emotional states^6,13,14^. In this study, a PPG and GSR recording device (HUAKE HK-2013/2GR-L) employing a fingertip clip on the index finger was used to acquire PPG signals, as shown in Fig. 1(b). The device communicated with the computer via Bluetooth. Data collection was performed using the manufacturer’s proprietary software, with the sensor operating at a sampling frequency of 200 Hz.

#### GSR

The GSR reflects variations in the skin’s electrical conductivity caused by sweat gland activity, which is regulated by the sympathetic nervous system. This physiological signal is extensively utilized in affective science and emotion studies^6,15,16^. The GSR signal was collected by the aforementioned PPG and GSR recording device, which indirectly measured skin conductance by detecting skin resistance. The sensor’s measurement range is 100 kΩ - 2500 kΩ, with a sampling frequency of 200 Hz.

### Experimental procedure

**Fig. 2:**
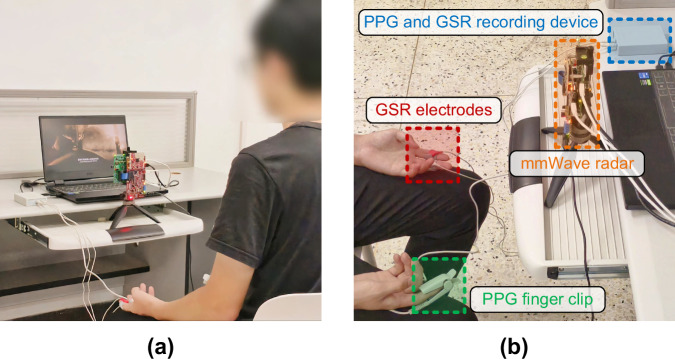
Experimental setup. (**a**) Overview. (**b**) Close-up of sensors placement.

The experiment was conducted in a laboratory. The film clips were played on a laptop computer placed on a desk. The clips were displayed in full screen mode on a 15.6-inch screen with a resolution of 1920 × 1080, and the audio was presented through the built-in speakers. The screen brightness and speaker volume were adjusted according to each participant’s preferences. The laptop was connected to the mmWave radar and the multimodal sensor device (recording PPG and GSR signals). Participants were seated in an upright chair to maintain consistent posture. To minimize motion artifacts, GSR electrodes were attached to the index and middle fingers of their non-dominant hand, while the index finger of the other hand wore a PPG finger clip. The mmWave radar was mounted vertically on a supporting stand and positioned at the edge of the desk, with the participant’s chest directly facing the radar antenna module. The distance between the radar and the participant’s chest varied across individuals, ranging from 0.6 m to 0.9 m, which better reflected real-world conditions. Figure 2 depicts a participant facing the mmWave radar while wearing PPG finger clip and GSR electrodes.

Prior to the data collection, the experimenter explained the experimental procedure and the use of SAM for subjective emotional ratings. The participant was also instructed to remain as still as possible while viewing the film clips to minimize motion artifacts. After fully understood, the participant signed the informed consent form. The experimenter then attached sensors to the participant and checked the signals. Given that sensors were attached to both of the participant’s hands, independent self-assessment after each clip was impractical. Therefore, the experimenter assisted in this process. Specifically, for each trial, once the participant was ready, the experimenter executed a Python script that sequentially triggered the sensors and played the clip. Immediately afterward, the experimenter quietly moved to a position outside the participant’s field of view. After a brief interval, the clip started playing, during which the experimenter remained silent. Upon the clip’s conclusion, the experimenter presented the SAM to the participant, who calmly reported their subjective ratings. The experimenter recorded the responses without engaging in further interaction. This concluded the current trial.

**Fig. 3:**
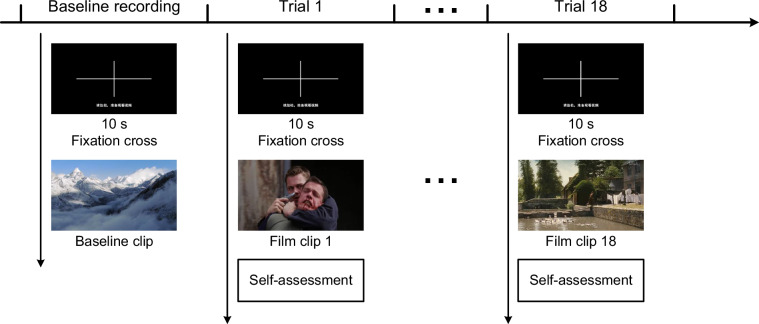
Experimental procedure flowchart.

As shown in Fig. 3, the experimental procedure comprised a baseline recording session followed by 18 trials. During the baseline recording, participant watched a documentary clip^12^. This baseline clip established the individual physiological baseline, allowed participant to adapt to the experimental environment, and facilitated emotional stabilization before the main trials. Before each clip, a 10-second fixation cross was displayed. This directed the participant’s attention, and provided an emotional reset period to minimize carryover effects from previous trials^17^. The process of a trial was as follows: (1) A 10-second fixation cross, (2) A film clip lasting 65–350 seconds^12^, (3) Subjective emotional ratings were assessed using three scales: valence, arousal, and dominance. Since the 18 clips contain 9 target emotions, with each target emotion corresponding to two clips, to avoid these two clips being played consecutively, which could lead to participant developing adaptation to the second clip and affecting the clip’s stimulus effect, the display order of the film clips for each participant was determined using constrained randomization. The constraint ensured that any two clips sharing the same target emotion were separated by at least two clips representing different emotions. The experiment duration is approximately 90 minutes.

## Data Records

The dataset^18^ collected in this study is available on Zenodo (10.5281/zenodo.15825931). The dataset comprises three components: raw data, processed data, and features. They are stored in “01_raw_data.zip”, “02_processed_data.zip”, and “03_features.zip”, respectively. Details are provided below.

### Raw data

**Fig. 4:**
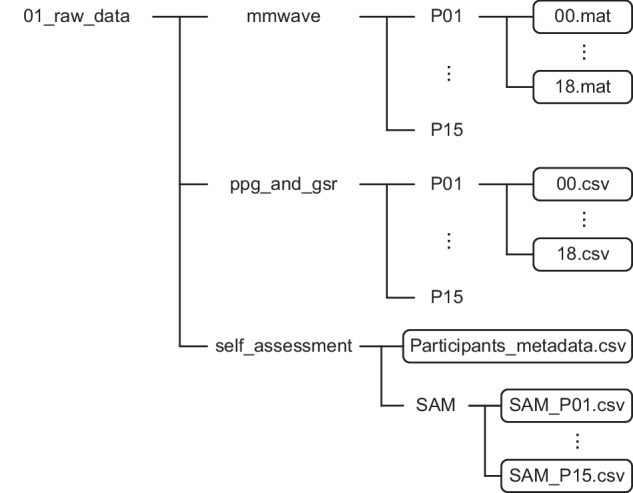
File directory structure of folder “01_raw_data”.

The raw data recorded by mmWave radar, PPG, and GSR sensors during participants’ viewing of film clips, along with the self-assessment results of each participant, are stored in the zipped file “01_raw_data.zip”, which contains three subfolders: “mmwave”, “ppg_and_gsr”, and “self_assessment”. The file directory structure is illustrated in Fig. 4.

The subfolder “mmwave” stores the mmWave radar raw data, organized by participant ID. Each participant’s folder includes.mat files corresponding to individual film clips, named by clip ID. Each.mat file contains a matrix named “adcData”, where the number of rows equals the product of the number of Tx antennas and Rx antennas, and the number of columns equals the product of ADC samples number, chirp loops number, frame rate, and the duration of the current clip (in seconds). In this study, a virtual antenna array consisting of 3 Tx and 4 Rx antennas was used, so the matrix has 12 rows, with each row representing data received by a virtual antenna. The data is arranged sequentially by chirp, meaning that after containing the ADC data points of one chirp, the data points of the next chirp follow.

The subfolder “ppg_and_gsr” stores the PPG and GSR raw data, organized by participant ID. Each participant’s folder includes.csv files corresponding to individual film clips, named by clip ID. The first row of each.csv file indicates the sampling frequency, and the second row identifies the data type. The first column contains PPG data while the second column contains GSR data, both sampled at 200 Hz. Notably, for all three types of collected data, each file only includes the period during which the participant viewed the corresponding clip, with irrelevant segments removed (the 10-second fixation cross before each clip is retained for future use).

The subfolder “self_assessment” stores the participants’ metadata and their self-assessment results. The metadata file is named “Participants_metadata.csv”, which records information such as each participant’s gender, age, dominant hand, experiment date, and the order in which clips were displayed. The subfolder “SAM” stores the participants’ SAM ratings in.csv format, with filenames formatted as “SAM_Pxx.csv”, where “xx” represents the participant ID. The first row of each.csv file is the header, the first column lists clip IDs, and the second, third, and fourth columns contain valence, arousal, and dominance ratings, respectively.

### Processed data

**Fig. 5:**
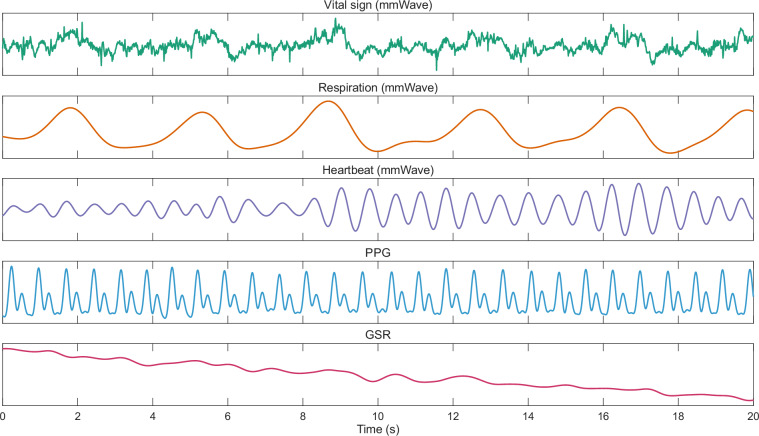
An example of the processed signals. The respiration and heartbeat are extracted from mmWave vital sign.

The processed data generated from the raw data are stored in “02_processed_data.zip”, organized into three subfolders: “mmwave”, “ppg”, and “gsr”. During processing, we removed the data corresponding to the first 10 seconds of the fixation cross in each trial. Figure 5 displays an example of the processed signals waveform. It is worth noting that this paper only illustrates a specific signal processing method as a demonstration. Users can apply different signal processing methods to the raw data in the dataset according to their needs.

In the “mmwave” subfolder, the file “mmwave_Pxx_yy.csv” contains the mmWave signals for participant “xx” during clip “yy”. The first row of the file is the header, with the first column representing the phase of vital sign, and the second and third columns containing the extracted respiratory and heartbeat signals, respectively. The workflow for deriving these signals from the raw mmWave data is as follows:

1. Signal model: The transmitted signal of an FMCW mmWave radar is characterized by a frequency-modulated continuous wave, commonly referred to as a chirp. During each chirp, the instantaneous frequency varies linearly with time. The transmitted signal and the received echo are combined in a mixer, which produces an intermediate frequency (IF) signal. The frequency and phase of this IF signal are determined as follows:
  1 $${f}_{IF}=S\tau (t),$$
  2 $${\phi }_{IF}(t)=\frac{4\pi R(t)}{{\lambda }},$$
  here, *S* denotes the frequency modulation slope of the chirp signal, defined as *S* = *B* / *T*_*c*_, where *B* is the chirp bandwidth and *T*_*c*_ is the chirp duration. The round-trip time delay of the signal is represented by *τ*, which corresponds to the target distance *R* relative to the radar. The wavelength of the mmWave radar is given by *λ* = *c* / *f*_*c*_, with *c* being the speed of light and *f*_*c*_ the starting frequency of the chirp. The time delay *τ* can be expressed as:
  3 $$\tau =\frac{2R}{c},$$
  by combining with Eq. (1), the fundamental principle of mmWave radar ranging can be obtained:
  4 $$R=\frac{c}{2S}{f}_{IF},$$
  from Eq. (2), the phase difference of the IF signal is related to the target displacement Δ*d* by:
  5 $$\Delta \phi =\frac{4\pi \Delta d}{\lambda },$$
  therefore, even the slight displacement of the human chest wall induces a significant phase change in the IF signal. This phase sensitivity enables the core physical mechanism for non-contact vital sign detection using mmWave radar.
2. Range-FFT: The IF signal from each receiving antenna is digitized to form a complex matrix. In this matrix, one dimension corresponds to the chirp index (slow time), and the other to the sampling points within a single chirp (fast time). A fast Fourier transform (FFT) is then performed along the fast-time dimension for each chirp. The resulting spectrum contains frequency components corresponding to different distances; the peak position in this spectrum identifies the intermediate frequency *f*_*IF*_, which is directly related to the target distance. From Eq. (4), where *c* is the speed of light and *S* is a known parameter, the distance *R* can be determined. Repeating this process for all chirps and arranging the spectra along the slow-time dimension yields a range-time matrix (RTM) with dimensions *N*_*range*_ × *N*_*chirp*_, where *N*_*range*_ is the number of range bins (equal to the fast-time samples per chirp) and *N*_*chirp*_ is the number of chirps (representing the slow-time dimension). This entire procedure is known as the Range-FFT.
3. Moving target indication: Objects in the environment, such as furniture and walls, also reflect radar signals. Therefore, moving target indication (MTI) is employed to suppress static clutter, specifically through mean cancellation^19^. Fundamentally, this approach relies on the distinct slow-time behaviors of static and moving targets. While echoes from stationary objects remain largely invariant within a given range bin, moving targets exhibit continuous phase and amplitude shifts. To suppress the stationary background, we first average the signals across all chirps to establish a baseline for the static clutter. Subtracting this mean from the raw data then filters out the static components, isolating the moving targets. For the RTM **X**, the mean value for the *i*-th range bin is computed as:
  6 $${\mu }_{i}=\frac{1}{{N}_{chirp}}\mathop{\sum }\limits_{j=1}^{{N}_{chirp}}{\bf{X}}(i,j),$$
  then, each sampling point in every range bin is subtracted by its corresponding mean value. After applying the mean cancellation to suppress static clutter, the value at the *i*-th range bin and the *j*-th chirp in the **X** becomes:
  7 $$\hat{{\bf{X}}}(i,j)={\bf{X}}(i,j)-{\mu }_{i}.$$
4. Multi-bin fusion: The vital sign, which includes respiration and heartbeat, is manifested in the phase along the slow-time dimension. Typically, the phase is extracted from the range bin corresponding to the target’s location. However, in practice, the target energy may spread across multiple adjacent range bins due to the limited range resolution, spectral leakage, or body movements. To fully utilize the signal energy and improve the robustness of phase estimation, we employ a multi-bin fusion method. First, identify the *K* range bins (*K* = 5 in this paper) with the highest average amplitudes across the slow-time dimension, indicating the bins containing significant target contributions. For each selected range bin *i*_*k*_ (*k* = 1, …, *K*), the phase is extracted:
  8 $${\phi }_{k}(j)={\rm{unwrap}}(\arctan (\hat{{\bf{X}}}({i}_{k},j))),\,j=1,2,\ldots ,{N}_{chirp},$$
  here, “arctan(·)” denotes the arctangent operation, which is performed on complex values to extract the phase. “unwrap(·)” refers to phase unwrapping. The true physical phase is continuous, but the computed phase is confined to a 2π interval, resulting in phase wrapping. Phase unwrapping recovers the true phase by applying a −2π shift when the phase change exceeds π, and a + 2π shift when the change is less than −π. To optimally combine the phases from multiple range bins, a weighted fusion strategy is employed. The weights are derived from the average amplitudes of the corresponding range bins:
  9 $${w}_{k}=\frac{A({i}_{k})}{{\sum }_{n=1}^{K}A({i}_{n})},\,k=1,\ldots ,K,$$
  where *A*(*i*_*k*_) is the average amplitude across slow time for range bin *i*_*k*_. The weights prioritize range bins with stronger signal energy, as they typically exhibit higher SNR. The fused phase is then computed:
  10 $${\phi }_{fused}(j)={\sum }_{k=1}^{K}{w}_{k}\cdot {\phi }_{k}(j),\,j=1,\ldots ,{N}_{chirp},$$
5. Vital signs extraction: For the *ϕ*_*fused*_ of each of the 12 virtual antenna elements, we take their average to perform multi-channel averaging to suppress noise and improve SNR. Low-frequency noise is removed using a first-order difference, and the resulting signal is regarded as the vital sign. A 4th-order Butterworth filter with a passband of 0.1–0.5 Hz is applied to extract the respiratory signal, while a 6th-order Butterworth filter with a passband of 1.0–1.8 Hz is used to extract the heartbeat signal.

In the “ppg” subfolder, the file “ppg_Pxx_yy.csv” contains the PPG signals for participant “xx” during clip “yy”. This file contains a single column of signal values without a header. The PPG signal processing involved filtering the raw signal with a 4th-order Butterworth filter (passband: 0.6–5 Hz)^20^, followed by a linear detrending process (subtracting the best straight-line least-squares fit from the data) to remove baseline drift. Finally, smoothing is performed using a moving average filter with a window size of 20 samples (equivalent to 0.1 seconds).

Similarly, in the “gsr” subfolder, the file “gsr_Pxx_yy.csv” contains the GSR signal for participant “xx” during clip “yy”. This file contains a single column of signal values without a header. Note that the raw GSR data was acquired in resistance units, so we first convert it to conductance. The processed GSR signal is obtained by applying a 3rd-order Butterworth low-pass filter with a cutoff frequency of 1 Hz^21^.

Regarding artifact handling for mmWave, PPG and GSR signals, we primarily rely on the aforementioned bandpass filters to mitigate high-frequency noise and low-frequency baseline fluctuations. We do not employ specific artifact rejection algorithms to remove motion-induced spikes, thereby preserving the temporal integrity of the signals for naturalistic emotion analysis.

### Features

We provide the features extracted from the three types of signals for emotion recognition, stored in “03_features.zip”. Detailed descriptions about these features and their extraction methods can be found in the Technical Validation section under Emotion recognition. The subfolders “mmwave”, “ppg”, and “gsr” contain the respective feature files, stored in.mat format. The naming convention “xxFea_Pyy_zz.mat” indicates the features of the “xx” signal for participant “yy” while watching clip “zz”. Each feature file contains a matrix named “featureMatrix”, where rows indicate the number of sliding windows (yielding 12 windows per clip, features are extracted from the final 60 seconds using a 5-second window with no overlap, as detailed in the “Emotion recognition” subsection), and columns represent the feature dimensions.

## Technical Validation

### Self-assessment analysis

Each participant completed self-assessment for all 18 film clips they viewed, rating each clip on three emotional scales: valence, arousal, and dominance. We first examine the coefficient of variation (CV) of participants’ ratings for each film clip. For a specific clip, the CV is defined as the ratio of the SD to the mean across all participants:

11 $$CV=\frac{\sigma }{\mu },$$

where *σ* is the SD and *μ* is the mean of the ratings for that clip. We then calculate the overall mean of these CVs across all 18 film clips. A smaller CV indicates stronger consistency among participants. The mean CV values with SDs for all participants are 0.26 ± 0.12 for valence, 0.32 ± 0.13 for arousal, and 0.29 ± 0.10 for dominance. These results demonstrate strong consistency in participants’ ratings across all three emotional scales, indicating that the emotions elicited by the film clips are relatively similar among different participants, which also validates the effectiveness of the stimuli.

**Fig. 6:**
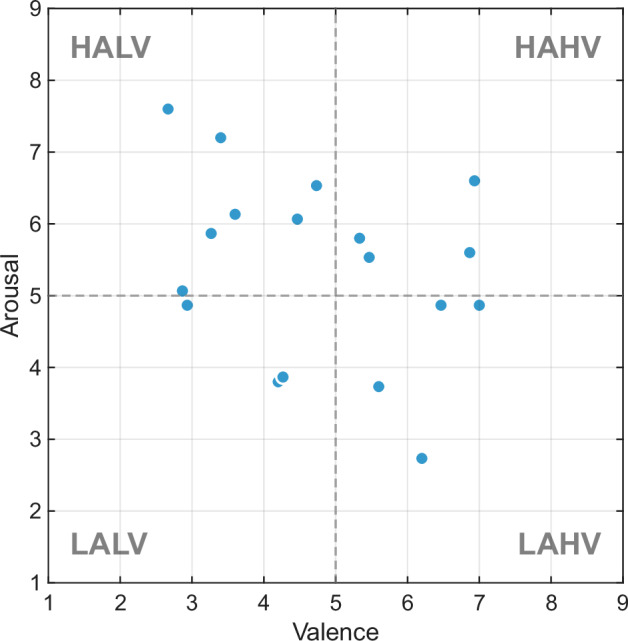
Distribution of clip ratings in valence-arousal space.

Table 3 presents the mean ratings and SDs of all film clips across the three emotional scales, while Fig. 6 displays the distribution of the clips’ ratings in the valence-arousal space. The valence-arousal space could be divided into four quadrants: low arousal low valence (LALV), low arousal high valence (LAHV), high arousal low valence (HALV), and high arousal high valence (HAHV). The results show that the average ratings of the 18 film clips successfully cover all four quadrants, indicating that these clips represent a comprehensive and diverse range of emotional categories. Additionally, the overall distribution of ratings partially aligns with the U-shaped pattern observed in the International Affective Picture System (IAPS) standard^22^, further supporting the evidence that the emotions elicited by the clips effectively reflect participants’ subjective experiences.

**Table 3:** Mean ratings and SDs for film clips across all participants.

Clip ID	Valence	Arousal	Dominance
1	6.87 ± 0.92	5.60 ± 1.68	6.67 ± 1.23
2	6.47 ± 0.99	4.87 ± 1.88	7.00 ± 0.76
3	4.73 ± 1.49	6.53 ± 1.30	6.13 ± 1.51
4	5.33 ± 1.18	5.80 ± 2.01	6.47 ± 1.19
5	6.93 ± 0.70	6.60 ± 1.30	5.40 ± 1.92
6	7.00 ± 1.00	4.87 ± 1.46	5.93 ± 1.53
7	6.20 ± 1.15	2.73 ± 1.75	6.93 ± 1.39
8	5.60 ± 0.74	3.73 ± 1.39	6.67 ± 1.40
9	3.27 ± 1.39	5.87 ± 1.36	4.53 ± 1.68
10	4.20 ± 1.08	3.80 ± 1.74	6.20 ± 1.47
11	5.47 ± 2.26	5.53 ± 1.55	5.40 ± 1.92
12	2.67 ± 1.40	7.60 ± 0.99	3.40 ± 1.18
13	3.60 ± 0.99	6.13 ± 1.73	4.27 ± 1.53
14	3.40 ± 0.91	7.20 ± 1.32	3.93 ± 1.91
15	2.87 ± 0.92	5.07 ± 1.58	4.67 ± 1.63
16	2.93 ± 1.03	4.87 ± 1.51	4.80 ± 2.11
17	4.27 ± 1.10	3.87 ± 2.03	6.07 ± 1.71
18	4.47 ± 1.13	6.07 ± 2.22	5.40 ± 1.64

The film clips used in this study are sourced from the research by Gabert-Quillen *et al*.^12^, enabling a comparison between our ratings and their established reference values. In this study, we utilize Spearman’s rank correlation coefficient (*ρ*) to assess the monotonic relationships between variables. Given the discrete nature of the SAM rating scales, tied values naturally occur. Therefore, Spearman’s *ρ* is strictly computed as the Pearson correlation coefficient between the rank variables, ensuring ties are handled correctly by assigning average ranks:

12 $$\rho =\frac{{\sum }_{i=1}^{n}(R({x}_{i})-{\bar{R}}_{x})(R({y}_{i})-{\bar{R}}_{y})}{\sqrt{{\sum }_{i=1}^{n}{(R({x}_{i})-{\bar{R}}_{x})}^{2}{\sum }_{i=1}^{n}{(R({y}_{i})-{\bar{R}}_{y})}^{2}}},$$

here, *R*(*x*_*i*_) and *R*(*y*_*i*_) denote the ranks of the paired variables *x* and *y*, ${\bar{R}}_{x}$ and ${\bar{R}}_{y}$ are their respective mean ranks, and *n* is the total number of paired observations. To assess consistency, we compute Spearman’s *ρ* for the mean valence and arousal ratings across the two studies. A higher coefficient indicates stronger correlation. Dominance ratings are not compared, as they were not recorded in the reference research. For valence, the *ρ* = 0.839, and for arousal, *ρ* = 0.904. The high correlation coefficients reflect a strong consistency in ratings between the two sets of ratings.

There may be potential correlations between different scales due to factors such as participant habituation and fatigue effects during the experiment^6^. Table 4 presents the Spearman’s rank correlation coefficients between the mean ratings of each emotion scale and the display order of the clips. The strongest correlation is observed between valence and dominance, exhibiting a significant, moderately strong positive relationship (*p < *0.001). While this does not imply causation, it suggests that when participants felt pleasant or joyful, they also tended to perceive greater emotional control. Conversely, when participants felt sad or distressed, they were more likely to experience their emotions as overwhelming and passive. This finding aligns with intuitive expectations and psychological theories^23^. Furthermore, significant, moderate positive correlations are found between valence and arousal, as well as between arousal and dominance (*p < *0.001). The intercorrelations among the three emotion scales indicate that they are not entirely independent. Notably, the clip display order is not significantly correlated with any scale, suggesting that habituation and fatigue effects were effectively mitigated.

**Table 4:** Correlations between valence, arousal, dominance ratings and clip order across all stimuli (t-test significance levels: *p < 0.05, **p < 0.01, ***p < 0.001).

Scale	Valence	Arousal	Dominance	Order
**Valence**	1	0.41***	0.47***	−0.07
**Arousal**		1	0.43***	0.06
**Dominance**			1	−0.03
**Order**				1

### Signal quality validation

#### PPG and GSR

We use SNR as an indicator to validate the quality of PPG and GSR signals. For PPG signals, a cutoff frequency of 15 Hz is employed as the boundary to distinguish between the meaningful signal and high-frequency noise^24^. Specifically, a 3rd-order Butterworth low-pass filter is applied to extract the PPG signal, while a 3rd-order Butterworth high-pass filter is applied to isolate the noise component. The SNR is then calculated using the equation, *SNR* = 10log_10_(*M*_*signal*_ / *M*_*noise*_), where *M*_*signal*_ and *M*_*noise*_ denote the mean square amplitudes of the low-pass filtered signal and the high-pass filtered noise, respectively^25^. For GSR signals, a 1 Hz cutoff frequency is used as the boundary, and similar operations are performed to calculate SNR^26^. The results show that for PPG signals, the mean SNR across all trials for each participant ranges from 48.16 dB to 53.06 dB, with SDs between 0.16 dB and 1.57 dB, median values ranging from 48.21 dB to 53.05 dB, and only 5% of the signals exhibiting an SNR below 47.27 dB. For GSR signals, the mean SNR across all trials for each participant ranges from 25.06 dB to 42.56 dB, with SDs between 0.67 dB and 6.26 dB, median values ranging from 26.45 dB to 42.36 dB, and only 5% of the signals exhibiting an SNR below 26.33 dB. The SNR values obtained in this study are comparable to those reported by Saganowski *et al*.^27^, which ranges from 26.66 dB to 37.73 dB. This consistency supports the reliability of our data acquisition and processing pipeline.

#### mmWave signal

The mmWave radar does not directly capture heartbeat signals, making it difficult to calculate SNR using the previously described method. In this study, we use PPG signals as a reference to validate mmWave signal quality by comparing heart rate (HR) errors between mmWave-derived heartbeat signals and PPG signals. First, heartbeat signals are extracted from the raw mmWave data, with the detailed process described in the Processed data section of the Data Records. Then, we identify the dominant frequency (i.e., HR) in both mmWave and PPG signals for each film clip. The results show that the mean absolute error (MAE) for individual participants ranges from 1.50 bpm to 4.44 bpm, with SDs ranging from 1.14 bpm to 5.85 bpm, and median absolute errors ranging from 1.10 bpm to 2.93 bpm. Across all participants, the MAE is 2.76 bpm, the median absolute error is 1.46 bpm, and the mean relative error is 3.53%. Although participants were instructed to remain as still as possible while watching the clips, factors such as fatigue and emotional responses to the clips led to random body movements. In this study we do not specifically remove signals corresponding to body movements, preserving data integrity while still obtaining relatively accurate HR measurements. Users interested in further refinement may process the data to eliminate body displacement artifacts as needed.

The objective of this study is to construct a dataset, the proposed method for heartbeat signal acquisition is a relatively basic approach. In the field of mmWave radar-based HR detection, numerous advanced algorithms already exist^28–32^. Readers seeking better performance are encouraged to explore those methods for further improvements.

### Emotion recognition

Although the film clips are originally selected based on nine discrete target emotions to ensure a diverse distribution across the affective space, in the emotion recognition task, participants’ subjective ratings of the clips are used as the ground truth labels. We focus on the dimensional model in this study; however, the discrete target emotion associated with each clip is available for users interested in categorical emotion recognition. For the ratings across the three emotion scales, we employ a threshold of 5, where scores below 5 are considered as “low” and scores above 5 as “high” on the respective scale. This threshold is selected because 5 represents the exact median and neutral point on the 9-point SAM scale. Binary classification based on this median is a standard and objective baseline approach widely adopted in emotion recognition research (e.g., the DEAP^6^ dataset). Alternatively, using two thresholds to create a three-level classification scheme (e.g., low, medium, and high) may allow for more detailed emotional differentiation. However, in this paper, introducing a “medium” category would reduce the number of samples per class, potentially worsening class imbalance and lowering overall classification performance. Therefore, we frame this task as a binary classification problem to establish a foundational baseline for validating the dataset’s utility, while leaving multi-class classification tasks for future research. The emotion recognition task comprises three sub-tasks, corresponding to participant-dependent binary classification for valence, arousal, and dominance respectively. This subsection evaluates the performance of emotion recognition using mmWave radar data and compares it with PPG and GSR for validation.

### Feature extraction

The details of processing raw signals from the three types of sensors have been described in the Processed Data section of the Data Records. In following sections, we will explain feature extraction.

For mmWave data, we extract a total of 32 features such as statistical, time-domain, frequency-domain and other relevant features^33–36^. To obtain heart rate variability (HRV) features from mmWave signals, individual heartbeat segmentation is required. In this study, we adopt the dynamic programming-based template matching algorithm proposed by Zhao *et al*.^37^. The code can be accessed through the resources provided in the Code Availability. For PPG signals, we extract 28 features similar to those of mmWave. As for GSR signals, following the methodology of Udovičić *et al*.^38^, we derive 24 features. The details are shown in Table 5. The specific HRV features extracted from both mmWave and PPG signals are defined as follows: meanNN (mean of normal-to-normal intervals), medianNN (median of normal-to-normal intervals), SDNN (SD of normal-to-normal intervals), RMSSD (root mean square of successive differences between adjacent normal-to-normal intervals), pNN50 (percentage of successive normal-to-normal intervals that differ by more than 50 ms), meanRate (mean heart rate), sdRate (SD of heart rate), HRVTi (HRV triangular index, representing the integral of the density distribution divided by the maximum of the density distribution), and SD1 (SD of the points perpendicular to the line of identity in the Poincaré plot, reflecting short-term HRV).

**Table 5:** Features of the signals.

Signal	Features
**mmWave**	Mean and SD of vital sign, mean absolute values of both first-order and second-order differences for vital sign and normalized vital sign, non-stationary index (NSI) and Higuchi fractal dimension (HFD) of vital sign, average energy of respiration signal power spectral density (PSD) within 0-0.l Hz, 0.1-0.2 Hz, 0.2-0.3 Hz and 0.3-0.4 Hz, average energy of heartbeat signal PSD within 1.0-1.3 Hz, 1.3-1.6 Hz and 1.6-1.8 Hz, mean instantaneous frequency and amplitude of the first four intrinsic mode functions (IMFs) derived from Hilbert–Huang transform (HHT) decomposition, HRV features: meanNN, medianNN, SDNN, RMSSD, pNN50, meanRate, sdRate and HRVTi and SD1 (Poincaré plot).
**PPG**	Mean and SD of signal, mean absolute values of both first-order and second-order differences for signal and normalized signal, NSI and HFD of signal, average energy of signal PSD within 1.0-1.3 Hz, 1.3-1.6 Hz and 1.6-1.8 Hz, mean instantaneous frequency and amplitude of the first four IMFs derived from HHT decomposition, HRV features: meanNN, medianNN, SDNN, RMSSD, pNN50, meanRate, sdRate and HRVTi and SD1 (Poincaré plot).
**GSR**	Median, mean, SD, minimum, maximum, and normalized range of signal, its first derivative, and its second derivative, median, mean, standard deviation, maximum, minimum and range of signal PSD within 0-2 Hz.

Given that some film clips are relatively long, participants might experience varied emotional responses as the plot developed. Moreover, certain emotions require temporal development—participants typically need exposure to the initial and middle segments of a clip to fully comprehend the contextual framework and storyline, during which their emotional states gradually evolve. In contrast, the latter segment of a clip is more likely to elicit pronounced emotional reactions. Therefore, we select the final 60 seconds of each clip for feature extraction^9^. For three types of signals, features are computed using a 5-second sliding window with a step size of 5 seconds. Based on the threshold established earlier, the distribution of participants’ samples across the three emotion scales is presented in Table 6.

**Table 6:** Distribution of all participants’ samples across the three emotion scales.

Emotion scales	Low samples	High samples	Total samples	High ratio
**Valence**	1344	1128	2472	45.63%
**Arousal**	960	1800	2760	65.22%
**Dominance**	1068	1860	2928	63.52%

### Emotion classification

Given that the primary contribution of this work is the construction and validation of a multimodal dataset rather than the development of novel machine learning methodologies, we employ the support vector machine (SVM), a classical classifier, to perform subject-dependent classification on emotional features as a fundamental baseline validation. The ground truth labels are derived from participants’ ratings on three emotional scales. For each trial, the first 75% of the samples are used as the training set, while the remaining samples serve as the test set^25^. For normalization, we apply Z-score standardization. Specifically, for each feature, the mean and SD are calculated across all samples in the training set. Then, each sample in both the training and test sets has its feature value subtracted by the mean and divided by the SD. In terms of model configuration, the SVM utilizes a radial basis function (RBF) kernel, with grid search enabled for hyperparameter tuning. The regularization parameter *C* and kernel coefficient *gamma* are selected from logarithmically spaced ranges: *C* from 11 values between 10^-5^ and 10^5^, and *gamma* from 6 values between 10^-4^ and 10.

Classification is performed on all three types of signals, and Table 7 presents their accuracy, balanced accuracy and F1-score results. It can be observed that for this dataset, in the task of emotion recognition—which involves a high degree of individual subjectivity—the accuracy of binary emotion classification using traditional machine learning on PPG, mmWave, and GSR signals ranges from 60% to 70%. This result is comparable to the baseline performance of classic datasets such as DEAP^6^ and AMIGOS^10^. To examine the impact of class imbalance on the classification results, we also report the balanced accuracy, which is the arithmetic mean of recall for each class. This metric prevents the model from achieving inflated accuracy by favoring the majority class. The results show that the balanced accuracy for different modalities and various emotion scales consistently exceeds 60%. This demonstrates that despite the presence of class imbalance, the models have learned effective emotional representations from the data. It further validates that the PPG, mmWave, and GSR signals in this dataset indeed contain information related to the self-assessment emotions. Additionally, to provide a more comprehensive evaluation of the dataset, we also compute the F1-score. As the harmonic mean of precision and recall, the F1-score offers a balanced measure between the two. The results show that the F1-scores are consistently around 60%, closely aligning with the balanced accuracy reported earlier. This consistency further corroborates the quality and reliability of the dataset.

**Table 7:** Average accuracy, balanced accuracy and F1-score of all participants for different signals.

Signal	Accuracy			Balanced accuracy			F1-score		
	Valence	Arousal	Dominance	Valence	Arousal	Dominance	Valence	Arousal	Dominance
**PPG**	62.3%	64.7%	65.8%	61.1%	60.2%	61.4%	60.8%	58.6%	60.0%
**mmWave**	62.0%	69.3%	67.2%	61.9%	63.9%	62.2%	60.8%	63.2%	60.7%
**GSR**	64.2%	67.2%	66.3%	62.9%	64.3%	64.9%	62.2%	62.7%	61.9%

In summary, the classification results demonstrate the quality of this dataset, confirming that the signals contain meaningful information related to the emotional states of the participants. Furthermore, while mmWave radar may not exhibit superior performance compared to traditional contact-based modalities, these findings underscore its feasibility and significant value as a privacy-preserving, non-contact solution for emotion recognition.

### Limitations

While this dataset introduces multi-modal signals—particularly highlighting the advantages of mmWave radar in providing a comfortable, non-intrusive, and privacy-protective approach to emotion recognition—several limitations regarding its practical application and our experimental design should be noted. First, the participant sample is relatively small and homogeneous, consisting of 15 healthy college students aged 19 to 25. Future studies should aim for larger, more demographically diverse populations to ensure better generalization. Second, the total experiment duration is approximately 90 minutes. Although we implement fixation crosses and a randomized clip order to mitigate carryover effects, potential participant fatigue or loss of concentration toward the end of the data collection remains a factor. Third, we do not control for or record participants’ prior exposure to the selected film clips. While our use of immediate post-trial SAM ratings ensures that the labels reflect actual emotions, prior familiarity with the movies might influence the intensity of the emotion responses.

For signal quality, while participants were instructed to remain still, random body movements inevitably occurred. We provide the raw mmWave data without specifically filtering out all body displacement artifacts to preserve data integrity; therefore, users may need to apply advanced motion artifact removal algorithms depending on their specific tasks. Finally, while we successfully validate the mmWave-derived heartbeat signals against PPG data, we do not employ a contact-based respiration sensor to provide a ground-truth reference for the extracted mmWave respiratory signals. Users should be aware of this absence of direct quantitative validation, although the larger physical displacement of the chest wall during breathing generally ensures a higher SNR in radar sensing compared to heartbeats.

## Usage Notes

The dataset^18^ is available through the Zenodo repository (10.5281/zenodo.15825931). It includes raw data, processed data, and features. While the raw data (“01_raw_data.zip”) is essential, the contents of “02_processed_data.zip” and “03_features.zip” can be regenerated by running the source code we provided (https://github.com/CJ20Project/mmWaveEmoRecDataset). The data formats (mat and csv) ensure compatibility with various tools for reading and analysis.

## Data Availability

The dataset^18^ is available on Zenodo (10.5281/zenodo.15825931), including raw data, processed data, and features. They are stored in “01_raw_data.zip”, “02_processed_data.zip”, and “03_features.zip”, respectively. The raw data recorded by mmWave radar, PPG, and GSR sensors during participants’ viewing of film clips, along with the self-assessment results of each participant, are stored in the zipped file “01_raw_data.zip”. The processed data from mmWave radar, PPG, and GSR sensors, which are derived from the raw data, are stored in “02_processed_data.zip”. We also provide the features extracted from the three types of signals for emotion recognition, stored in “03_features.zip”. For detailed information about the dataset, please refer to the Data Records section.

## Code availability

The source code related to data collection is available at (https://github.com/CJ20Project/mmWaveEmoRecDatasetDataCollection), including the code for generating clip display sequences and launching the experimental trial. All source code is implemented in Python. More details can be found in the project’s README.md.

The source code for signal processing and the Technical Validation section of this study is available at (https://github.com/CJ20Project/mmWaveEmoRecDataset). To use the code, clone the repository and place the dataset folder in the data directory. The source code is implemented in MATLAB and Python. For details on the directory structure, environment setup, and code documentation, please refer to the project’s README.md file.
